# 

**Published:** 1889-06

**Authors:** 


### Bristol Medico-Chirurgical Journal.

### JUNE, 1889.

### CONTENTS.

Original Papers: *page*

Observations on the Normal Diet. By G. Munro Smith ... ... ... 73

On the Treatment of Laryngeal Growths. By Barclay J. Baron, M.B. Edin. ... ... ... ... ... ... ... ... ... ... ... ... 78

Clinical Records:

On "Birth Palsy," with Illustrative Cases. By F. W. Jollye, M.R.C.S., L.R.C.P., Warminster ... ... ... ... ... ... ... 87

Cases of Oral Surgery. By Nelson C. Dobson, F.R.C.S. ... ... ... 108

Health-Resorts:

Clifton. By John Beddoe, M.D., F.R.S., etc. ... ... ... ... ... ... ... 114

Reviews of Books ... ... ... ... ... ... ... ... ... ... ... ... ... ... 123

Notes on Preparations for the Sick ... ... ... ... ... ... ... ... 131

Periscope ... ... ... ... ... ... ... ... ... ... ... ... ... ... ... 134

The Visit of the British Medical Association to Bristol in 1891 ... 142

Scraps ... ... ... ... ... ... ... ... ... ... ... ... ... ... ... ... 143

